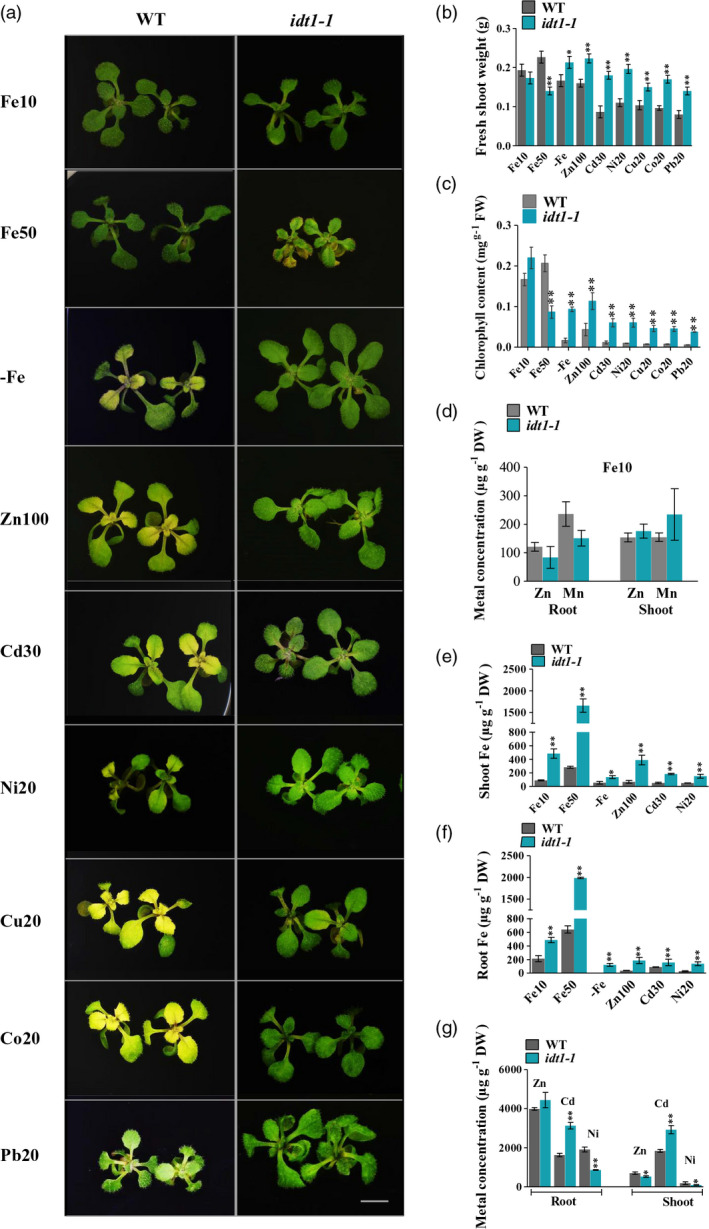# Corrigendum

**DOI:** 10.1111/pbi.13537

**Published:** 2021-02-08

The authors of Sharma and Yeh (2019) have identified an error in Figure 2a after publication. In the published version, some subfigures in Figure 2a were accidentally duplicated. In the left column, the subfigures of Cd30 and Ni20 were duplicated in the sets of Co20 and Cu20, respectively. However, the quantitative data is correct.

The amended figure is shown below.